# Primary thromboprophylaxis for cancer patients with central venous catheters – a reappraisal of the evidence

**DOI:** 10.1038/sj.bjc.6602917

**Published:** 2006-01-10

**Authors:** M S Cunningham, B White, D Hollywood, J O'Donnell

**Affiliations:** 1 Department of Haematology, Institute of Molecular Medicine, St. James's Hospital and Trinity College, Dublin, Ireland; 2 Academic Unit of Clinical and Molecular Oncology, Institute of Molecular Medicine, St. James's Hospital and Trinity College, Dublin, Ireland

**Keywords:** central venous catheter, malignancy, thrombosis, antithrombotic prophylaxis

## Abstract

Venous thromboembolism (VTE) is responsible for an estimated 25 000 deaths per annum in UK hospital practice. It is well established that many of these deaths could be prevented through the use of appropriate thromboprophylaxis. This issue is of particular relevance in oncology practice, where the risks of VTE and bleeding are both significantly higher than those observed in general medical patients. Cancer patients with in-dwelling central venous catheters (CVCs) are at particularly high risk of developing thrombotic complications. However, the literature has produced conflicting conclusions regarding the efficacy of using routine primary thromboprophylaxis in these patients. Indeed such is the level of confusion around this topic, that the most recent version of the American College of Chest Physicians (ACCP) guidelines published in 2004 actually reversed their previous recommendation (published in 2001). Nevertheless, minidose warfarin continues to be routinely used in many oncology centres in the UK. In this article, we have performed a systematic review of the published literature regarding the efficacy and the risks, associated with using thromboprophylaxis (either minidose warfarin or low-dose LMWH) in cancer patients with CVC. On the basis of this evidence, we conclude that there is no proven role for using such thromboprophylaxis. However, asymptomatic CVC-related venous thrombosis remains common, and further more highly powered studies of better design are needed in order to define whether specific subgroups of cancer patients might benefit from receiving thromboprophylaxis.

In the developed world, venous thromboembolism (VTE) develops in approximately one in 1000 people each year (Oger, 2000). It typically presents as a deep vein thrombosis (DVT) of the calf, which may extend proximally into the veins of the thigh. These proximal DVT can then give rise to pulmonary embolism (PE) (Kearon, 2003). Studies have demonstrated that PE are directly responsible for approximately 10% of all hospital in-patient deaths, and that they contribute to a further 15% (Geerts *et al*, 2004). Consequently, over 25 000 people are estimated to die from VTE each year in England alone, and clinical management of acute VTE costs the UK in excess of £600 million per annum.

It is well recognised that patients with cancer constitute a particularly high-risk group for both arterial and VTE. Compared to age and sex-matched controls, the relative risk of VTE is increased approximately five-fold in patients with cancer (Heit *et al*, 2000). Clinically symptomatic DVT have been reported in up to 15% of patients with cancer (Bick, 1978). However postmortem studies have demonstrated asymptomatic VTE in as many as 50% (Ambrus *et al*, 1975). Multiple mechanisms have been implicated in explaining the increased incidence of VTE associated with cancer (Piccioli *et al*, 1996). Moreover, it is well established that specific therapeutic interventions (including surgery, chemotherapy or hormone-based treatment) can further increase the absolute risk of VTE (Lee, 2005).

Central venous catheters (CVCs) were initially developed by Broviac *et al* in 1973, (Broviac *et al*, 1973) and then subsequently modified by Hickman *et al* (1979). They are now widely used in cancer patients who require intensive chemotherapy and/or stem cell transplantation. These venous access devices are typically in the form of either an external catheter (e.g. Hickman or Groshong line), or an implanted subcutaneous port (e.g. Port-a-Cath). Although central venous access has revolutionised the clinical management of cancer patients, there is strong evidence to suggest that both catheters and ports are associated with a significant increase in the rate of VTE. The aetiology of CVC-related thrombosis is likely to be multifactorial in origin. Insertion of CVCs is associated with traumatic damage to the vessel wall, and further damage to the endothelial cell lining of the vessel wall may occur depending upon the final position of the line tip (Eastridge and Lefor, 1995). In addition, total parenteral nutrition, chemotherapy and other drugs infused through the CVC may exacerbate any local areas of vessel wall damage (Baglin and Boughton, 1986). Cancer patients with constitutional thrombophilias (notably antithrombin deficiency) appear to be at an increased risk of CVC-related thrombosis, (De Cicco *et al*, 1995; Tesselaar *et al*, 2004) as do those with elevated homocysteine levels, but not those with the factor V Leiden or prothrombin 20210A gene mutations (De Cicco *et al*, 1995; Tesselaar *et al*, 2004). Ovarian carcinoma has also been associated with a higher incidence of CVC-related venous thrombosis compared to other tumour types (Tesselaar *et al*, 2004).

The high rate of VTE associated with CVC in cancer patients has led to the suggestion that all such patients should receive primary thromboprophylaxis (in the form of either heparin or warfarin). Indeed the practise of using thromboprophylaxis for cancer patients with CVC is now considered routine practise in many oncology centres across the UK. However, it is well established that heparin or warfarin use in cancer patients is associated with an increased risk of bleeding. Consequently the appropriate role of primary thromboprophylaxis in cancer patients with CVCs represents a controversial area. In order to establish whether the available evidence supports current practise, we have performed a systematic review of the published literature on the efficacy and the risks associated with using thromboprophylaxis (either minidose warfarin, or LMWH), in cancer patients with indwelling CVC.

## METHODS

### Search strategy

Data for this review was identified by searching the electronic databases MEDLINE, EMBASE and the Cochrane Library (encompassing period January 1966 until November 2005) by two independent reviewers (MC, JOD). The search terms were ‘catheterisation, central venous’, ‘thrombosis’, ‘oncology’, ‘neoplasms’, ‘adult’, ‘anticoagulation therapy’ and ‘haematological’ as Medical Subject Heading (MeSH) terms and text words. In addition, the bibliographies of all retrieved articles were also manually searched for additional relevant articles. Only full papers published in English between 1980 and 2005 were considered.

### Selection criteria

Studies were included in the review if they met the following criteria:

- Prospective design.
- Study population consisted of adult (more then 14 years) cancer patients, receiving a CVC – defined as either implantable port or external catheter.
- Site of catheter insertion defined.
- Some or all of the patients received either heparin or warfarin thromboprophylaxis at clearly specified dose.

For most articles, selection criteria were apparent. For articles in which criteria were unclear, the decision for selection was made by consensus among all the authors.

### Data extraction and synthesis

Each study fulfilling the inclusion criteria was reviewed independently, by two or more of the authors. All data were extracted according to a predetermined standard checklist. Disagreements were resolved by consensus among the authors. The methodologic quality of each study was critically examined. Clinical outcomes of interest included incidence of asymptomatic and/or symptomatic DVT, PE, and overall mortality. In view of the nature of the data retrieved, quantifiable analysis (met-analysis) was not performed as part of this study.

## RESULTS

Using the specified selection criteria, a total of nine prospective studies of thromboprophylaxis use in adult cancer patients with CVC were identified (Table 1) (Bozzetti *et al*, 1983; Bern *et al*, 1990; Monreal *et al*, 1996; Nightingale *et al*, 1997; Boraks *et al*, 1998; Heaton *et al*, 2002; Mismetti *et al*, 2003; Couban *et al*, 2005; Verso *et al*, 2005). Of these studies, one investigated unfractionated heparin thromboprophylaxis, (Bozzetti *et al*, 1983) while another three studied the efficacy of different low molecular weight heparin preparations (dalteparin, nadroparin and enoxaparin, respectively) (Monreal *et al*, 1996; Mismetti *et al*, 2003; Verso *et al*, 2005). Use of minidose warfarin (1 mg daily) was studied in six independent trials, incorporating 1463 patients (Bern *et al*, 1990; Nightingale *et al*, 1997; Boraks *et al*, 1998; Heaton *et al*, 2002; Mismetti *et al*, 2003; Couban *et al*, 2005).

### Incidence of catheter-related thrombosis in cancer patients

In the general population, upper limb DVT is uncommon, accounting for only approximately 2% of all episodes of DVT (Marie *et al*, 1998; Marinella *et al*, 2000). Long-term indwelling CVCs appear to be the most common predisposing factor for upper limb DVT, and have been implicated in between 22 and 72% of cases involving noncancer patients (Marie *et al*, 1998; Marinella *et al*, 2000). The actual incidence of CVC-related thromboses reported in cancer patients without thromboprophylaxis varies widely across different prospective studies (Table 1), and is highly dependent upon the nature of the study design. In particular, marked differences relate to whether clinical symptomatic thrombosis or asymptomatic thrombosis (detected only by venogram screening), are used as the endpoint of the study. It is also important to note that the incidence of both symptomatic (1–8%) and asymptomatic (12–18%) CVC-associated VTE reported in more recent studies (Mismetti *et al*, 2003; Couban *et al*, 2005; Verso *et al*, 2005) is also significantly lower than that reported in the older studies (symptomatic VTE 0–25%; asymptomatic VTE 27–62%) (Bozzetti *et al*, 1983; Bern *et al*, 1990; Monreal *et al*, 1996). This important observation may reflect improvements in the biocompatibility of the CVCs, and/or improvements in CVC insertion techniques.

In the prospective studies included in this review, two studies investigated the risk of venous thrombosis specifically associated with the use of implantable ports in cancer patients not receiving thromboprophylaxis. In a prospective follow-up study of 40 cancer patients with Port-a-Cath subclavian venous catheters who did not receive any thromboprophylaxis, Bern *et al* (1990) observed 15 (37.5%) venogram-proven DVT (10 of which were symptomatic) after 90 days. In this small study, the incidence of thrombosis was not influenced by either the age of the patient, or by the tumour type. In a similar study, Monreal *et al* (1996) identified catheter-related VTE by venography in 8/13 (62%) cancer patients with Port-a-Caths in the absence of prophylaxis.

Although several retrospective studies have suggested that the risk of thrombosis associated with external catheters may be significantly greater than that observed with implantable ports, this question has not been addressed in a prospective study. In terms of the different external catheters used in clinical oncology practise, the risk of thrombotic complications appears equivalent for both Hickman and Groshong catheters (Eastridge and Lefor, 1995). However, various other factors have been reported to influence the incidence of catheter-related thrombosis. For example, the risk of VTE is significantly higher in cancer patients who receive triple-lumen compared to double-lumen catheters and is higher if the catheter is inserted into the left rather than right subclavian vein (Gould *et al*, 1993; Craft *et al*, 1996). Furthermore, increased incidence of thrombosis has also been observed in cancer patients where the final catheter tip position has been placed above the T3 level (Eastridge and Lefor, 1995).

### Warfarin thromboprophylaxis in cancer patients with CVC

In view of the high risk of VTE associated with using indwelling CVC in cancer patients, several reviews have advocated the use of primary thromboprophylaxis (Klerk *et al*, 2003). However, it is well established that the risks of bleeding are significantly higher in cancer patients (Krauth *et al*, 1987; Bona *et al*, 1997). The risk of major bleeding associated with warfarin therapy can be reduced, by lowering the target INR. Studies have also shown that even low-dose warfarin (1 mg day^−1^) can reduce the rate of thrombin generation *in vivo*, (Bauer and Rosenberg, 1987) and significantly reduce the incidence of DVT following gynaecologic surgery (Poller *et al*, 1987). Furthermore, in patients with stage IV breast cancer, minidose warfarin (adjusted to maintain a target INR 1.3–1.9), was effective in reducing lower limb DVT (Levine *et al*, 1994). On the basis of these observations, several groups have studied the efficacy of minidose warfarin in cancer patients with CVC.

In an open randomised trial, Bern *et al* (1990) enrolled 82 cancer patients who required central venous access (Port-a-Cath subclavian catheters). The patients were then randomised to receive, or not to receive, warfarin (1 mg daily), beginning 3 days before CVC insertion and continuing for a further 90 days. Prothrombin times (PT) were monitored regularly throughout the study, and all patients were subsequently screened by venogram. Of the 42 patients randomised to receive low-dose warfarin therapy, four (9.5%) patients developed DVT. Conversely, in the control group of patients not receiving warfarin, 15 out of 40 (37.5%) patients were found to have developed venogram-proven DVT during the 90-day follow-up period (*P*<0.001). Despite this marked therapeutic efficacy, the use of low-dose warfarin did not significantly influence the PT in the majority of the patients studied, and was not associated with any increase in bleeding.

Minidose warfarin thromboprophylaxis has also been used as thrombophylaxis to prevent CVC-related thrombosis in patients with haematological malignancies. Boraks *et al* (1998) prospectively treated 108 consecutive patients with warfarin 1 mg day^−1^ commenced on the day of line insertion, and then continued until the catheter was removed. The prothrombin time was measured three times per week, and the warfarin dose adjusted to maintain a target INR <1.6. All patients presenting with clinical signs suggestive of DVT were investigated with Doppler ultrasound and/or venogram. Of the patients receiving warfarin prophylaxis, 5% developed clinically symptomatic DVT. The use of minidose warfarin was not associated with any increased bleeding complications. However, four patients were noted to have prolonged PT (>20 s) that necessitated temporary cessation of their warfarin therapy. Unfortunately this study did not include a prospective control cohort. Rather the effect of minidose warfarin was compared to a historical control group, which fails to satisfy the selection criteria of this systematic review.

Although the study of Bern *et al* demonstrated significant efficacy for minidose warfarin thromboprophylaxis in cancer patients, more recent studies have failed to reproduce these findings. Heaton *et al* (2002) investigated 88 adult patients with haematological malignancies who required insertion of either a double lumen Hickman line (*n*=78) or double lumen Groshong catheter (*n*=10). A total of 45 patients were randomised to receive warfarin 1 mg daily started on the day of insertion and continued for a further 90 days or until development of clinical thrombosis. The INR was monitored on a weekly basis, and if greater than 1.5, warfarin therapy was temporarily discontinued. In patients with clinical findings suggestive of either catheter thrombosis (difficulty in aspirating or injecting into the line), or DVT (arm pain or swelling), a venogram was performed. There were eight (18%) cases of confirmed thrombosis in the warfarin-treated group (six patients with catheter-related thrombosis and two patients with DVT), as opposed to five (12%) cases in the control group (four patients with catheter-related thrombosis and one patient with DVT), leading the authors to conclude that minidose warfarin was of no therapeutic benefit in preventing CVC-related thrombosis in patients with haematological malignancies. However, despite the original study design, it is noteworthy that the patients randomised to the warfarin treatment arm of this study only received warfarin therapy for a median 41 days. This reduced warfarin compliance related to a variety of factors including elevated INR, severe thrombocytopenia, and bleeding complications in one case. In all, 10 patients treated with warfarin developed prolonged INR >1.5, compared to five patients with no warfarin treatment. One patient on warfarin and INR >1.5 developed haematuria. However, this patient had Non-Hodgkin's lymphoma with tumour involving his ureters, and was also uraemic.

More recently, a larger randomised, double-blind, placebo-controlled trial of 255 patients with solid tumours and haematological malignancies also failed to demonstrate any reduction in the incidence of symptomatic venous thrombosis for patients treated with minidose warfarin. Couban *et al* (2005) randomised patients to receive either warfarin 1 mg daily or placebo. Therapy was commenced within 72 h of CVC insertion, and continued until the catheter was removed. Ultrasound and venography were only performed in patients presenting with symptoms suggestive of venous thrombosis. In total, there were 11 symptomatic CVC-related thrombotic events – five (4%) in the placebo group and six (4%) in the warfarin arm, respectively. The explanation for the conflicting conclusions reached by these studies may well relate to important differences in study design (most notably for example, Bern *et al* performed routine screening venography). In addition, although Bern's cohort consisted mainly of patients with solid tumours and lymphomas, the cohort enrolled in the study of Couban predominantly suffered from haematological malignancies.

### LMWH thromboprophylaxis in cancer patients with CVC

Warfarin therapy in cancer patients is associated with a number of important clinical issues. In particular, fluctuations in INR on equivalent doses of warfarin are a common problem due to GI disturbances (vomiting, diarrhoea), cachexia, liver disease and chemotherapy. In addition, warfarin therapy has a delayed onset of action, with the full anticoagulant effect not being reached until 2–3 days following commencement of treatment. This delayed onset of anticoagulant effect, together with the long half-life of warfarin, means that any surgical interventions must be carefully planned in cancer patients maintained on warfarin.

In view of the problems associated with use of warfarin in oncology patients, recent studies have investigated the role of LMWH thromboprophylaxis in cancer patients with CVC. In an open, prospective study, Monreal *et al* (1996) studied 29 cancer patients who were having Port-a-Caths inserted. In all, 16 patients were randomised to receive LMWH (Dalteparin 2500IU s.c. once daily, starting 2 h prior to insertion of CVC and continuing for 90 days). Venography was performed after 90 days or sooner if VTE symptoms were observed. The study was terminated early on the recommendation of the institutional review committee, as DVT developed in only 6% of the LMWH treatment group as opposed to 62% of the controls group (*P*=0.002). The use of LMWH was not associated with any increase in bleeding.

More recently, Verso *et al* reported the first randomised, double-blind, placebo-controlled study, to assess the efficacy and safety of enoxaparin thromboprophylaxis (Verso *et al*, 2005). A total of 385 cancer patients (including those with haematological malignancy) were randomised to either enoxaparin (40 mg subcutaneously once daily) or placebo, commencing 2 h before CVC insertion and continued for 6 weeks. All patients underwent screening venography performed 6 weeks after randomisation, which demonstrated venous thrombosis in 22 cancer patients (14%) treated with enoxaparin, compared to 28 cases 18%) treated with placebo, respectively. The authors concluded that LMWH thromboprophylaxis with enoxaparin was not associated with any significant reduction in CVC-associated venous thrombosis. In keeping with other recently reported studies, it is important to note that the rate of asymptomatic CVC-associated thrombosis in the placebo arm of this trial (18%) was significantly lower than that reported in the older studies (38 and 62%) (Bern *et al*, 1990; Monreal *et al*, 1996) In view of this reduction in absolute baseline thrombotic complications, future studies will need to be more highly powered in order to detect subtle differences with introduction of thromboprophylaxis.

The relative efficacies of LMWH compared to minidose warfarin have been compared in only one study. Mismetti *et al* (2003) randomised 59 cancer patients to receive either minidose warfarin (1 mg day^−1^) or LMWH (nadroparin 2850IU OD). Over the course of a 90-day follow-up, the incidence of venogram-proven upper extremity DVT was 17% in the warfarin-treated group compared to 29% for the nadroparin group (*P*=0.48). In this study, one patient with lung cancer receiving nadroparin developed fatal haemoptysis. On the basis of this small pilot, the authors concluded that there was no significant difference in efficacy between minidose warfarin and LMWH thromboprophylaxis.

## DISCUSSION

Patients with cancer are at increased risk of VTE, and placement of CVC further increases this risk. The development of upper extremity venous thrombosis in a cancer patient with an indwelling CVC constitutes an important clinical complication. Pulmonary embolism is generally reported to occur in 10–20% of patients with upper limb DVT (Horattas *et al*, 1988; Horne *et al*, 1995). However, in cancer patients with CVC-related thrombosis, the risk of PE is significantly higher, occurring in 15% of cases even if managed using therapeutic anticoagulation (Monreal *et al*, 1994). Consequently, the development of objectively proven upper limb venous thrombosis usually necessitates CVC removal. Subsequently another CVC will need to be inserted at another site. However, even the temporary loss of central venous access can result in significant clinical problems. Furthermore, following the objective diagnosis of any catheter-related DVT, cancer patients will require full-dose therapeutic anticoagulation (with heparin and/or warfarin) with the attendant risks of bleeding. Previous studies have also suggested an increased incidence of CVC sepsis in patients who develop CVC-related thrombosis (Klerk *et al*, 2003). In the longer term, post-thrombotic syndrome has been reported in 30% of cancer patients following upper limb DVT, and permanent venous damage can result from CVCs (approximately 30% cases), even in cancer patients without a history of CVC-related thrombosis (Kerr *et al*, 1990).

In this context, the question of whether primary thromboprophylaxis should be considered for cancer patients with CVC is clearly of direct clinical importance. However, as we have demonstrated in this systematic review, there is a paucity of evidence upon which to base clinical decision making regarding the role of thromboprophylaxis. In total, only nine prospective studies investigating the role of thromboprophylaxis in adult patients with cancer have been identified, and only within the last year have the first double-blind, randomised-controlled studies been published. Moreover, six of these nine studies enrolled fewer than 125 cancer patients in total (Bozzetti *et al*, 1983; Bern *et al*, 1990; Monreal *et al*, 1996; Boraks *et al*, 1998; Heaton *et al*, 2002; Mismetti *et al*, 2003). In view of the small numbers of patients in these studies, it is not perhaps surprising that they reached conflicting conclusions. Furthermore, as a consequence of the lack of patient numbers, stratification for important confounding factors (e.g. antithrombin deficiency; elevated homocysteine levels; side of CVC placement) has not been possible. In addition, the patient cohorts enrolled in the different studies demonstrate marked differences. For example, they include a wide variety of different tumour types and stages. Moreover, in only some of the studies were patients with haematological malignancies included, together with patients with solid tumours. Clearly, larger randomised studies are required in order to establish both the efficacy and the safety of thromboprophylaxis in cancer patients with CVCs.

The problem of small cohort size, and discrepant populations of patients, makes it difficult to perform any meaningful comparisons between studies in the form of conventional meta-analysis, or even systematic review. This is compounded by the fact there are critical differences in study design. For example, the wide range in the incidence of thrombotic events reported is highly influenced by whether the end point of the study was defined as symptomatic or asymptomatic thrombosis. In addition, some of the studies failed to make any distinction between DVT, and thrombosis restricted to the lumen of the catheter. Finally, interpretation of the data is further complicated by the fact that a significant minority of patients enrolled in these studies were unable to complete their assigned period of thromboprophylaxis. For example, in the study of Heaton *et al* (2002), 38% of lines had to be removed before 90 days in patients receiving warfarin due to line infection, and other causes besides thrombosis.

Notwithstanding the limitations discussed above, several conclusions can be drawn from this systematic review of the literature. Firstly, as previously discussed, the rate of CVC-related venous thrombosis is significantly lower in recent compared to older studies. In particular, symptomatic upper extremity DVT is now relatively unusual, occurring in only approximately 5% of cancer patients. In contrast, asymptomatic VTE is still detectable in around 20% cases. The clinical significance of such asymptomatic thrombi in patients with cancer remains unclear, as little is known regarding the natural history of CVC-related DVT. Further studies addressing this issue would be of clinical value.

Secondly, on the basis of the available data, it is not possible to recommend using routine thromboprophylaxis for all cancer patients receiving CVCs. In particular, it is important to note that the only two placebo-controlled trials performed to date failed to demonstrate any beneficial effect for either warfarin or LMWH (enoxaparin) thromboprophylaxis (Couban *et al*, 2005; Verso *et al*, 2005). However, it remains unclear how therapeutic efficacy and safety might vary over a range of different warfarin or LMWH doses. For example, dose of LMWH has been shown to be of critical importance in determining efficacy in cancer patients undergoing abdominal surgery (Carr and Rabinowitz, 2000). In addition, the future use of LMWH thromboprophylaxis may be influenced by accumulating recent literature suggesting that use of LMWH in cancer patients is associated with improved survival (Altinbas *et al*, 2004; Kakkar *et al*, 2004; Klerk *et al*, 2005; Lee *et al*, 2005). This survival benefit is not entirely explained by a reduction in symptomatic VTE, raising the intriguing possibility that LMWH may also exert a direct effect on tumour cell biology.

Finally, from the studies included in this review, it is clear that thromboprophylaxis with either warfarin or LMWH is generally well tolerated in cancer patients with indwelling CVC. Indeed thromboprophylaxis appears to be safe, even in patients with haematological malignancies who often have concurrent severe thrombocytopenia (Boraks *et al*, 1998; Cortelezzi *et al*, 2005). However, it is important to recognise that minidose warfarin (1 mg day^−1^) can have a significant effect on the prothrombin time in cancer patients (Heaton *et al*, 2002; Masci *et al*, 2003; Mismetti *et al*, 2003; Magagnoli *et al*, 2005). The aetiology of this increased prothrombin time is multifactorial, and includes anorexia and liver metastases (Magagnoli *et al*, 2005). In addition, it is well recognised that warfarin can interact with other medications, leading to significant increases in INR levels. In particular, several studies have demonstrated an adverse reaction between warfarin and fluorouracil (FU) (Masci *et al*, 2003; Magagnoli *et al*, 2005). Masci *et al* (2003) observed significant elevation of the INR in 33% of patients receiving the combination of minidose warfarin and FU. Similarly, Magagnoli *et al* (2005) demonstrated that concomitant use of minidose warfarin and the FOLFOX regimen (FU, folinic acid and oxaliplatin) resulted in INR elevation in 40% patients (range 1.5–9.4; median 2.9). Consequently, it is evident that minidose warfarin can significantly prolong the prothrombin time, at least in selected subgroups of cancer patients. On this basis, it would appear prudent that all cancer patients treated with minidose warfarin should have routine prothrombin time (INR) monitoring, and have their dose of warfarin titrated to maintain an INR ⩽1.5. To date there is no evidence to suggest that anti-Xa monitoring is indicated for cancer patients receiving prophylactic dose LMWH thromboprophylaxis.

## Figures and Tables

**Table 1:** Description of the prospective studies on thromboprophylaxis for cancer patients with CVC included in this review

**Study**	***n***	**Catheter**	**Location**	**Intervention**	**Duration**	**Test**	**Objective VTE**	**Symptomatic VTE**
Bozzetti *et al* (1983)^15^ (cohort study)	52	External	Subclavian	Heparin 2500–3100 U daily	6–38 days	Venogram	Heparin 5/15 (33%) Control 10/37 (27%)	Heparin 0/15 (0%)
Bern *et al* (1990)^16^ (open randomised study)	121	Port	Subclavian	Warfarin 1 mg daily	90 days	Venogram	Warfarin 4/42 (10%) Control 15/40 (38%)	Warfarin 4/42 (10%) Control 10/40 (25%)
Monreal *et al* (1996)^17^ (open randomised study)	32	Port	Subclavian	Fragmin 2500 IU s.c. once daily	90 days	Venogram	Fragmin 1/16 (6%) Control 8/13 (62%)	Not specified
Nightingale *et al* (1997)^18^ (cohort study)	832	External	Sublavian+ jugular+femoral	Warfarin 1 mg daily	Mean 122 days	Ultrasound± venogram	Symptomatic only	Warfarin 42/949 (4%)
Boraks *et al* (1998)^19^ (cohort study)	108	External	Subclavian	Warfarin 1 mg daily	Catheter duration	Ultrasound± venogram	Symptomatic only	Warfarin 5/108 (5%) (historical controls)
Heaton *et al* (2002)^20^ (open randomised study)	88	External	Subclavian	Warfarin 1 mg daily	90 days	Venogram	Warfarin 8/45 (18%) Control 5/43 (12%)	Not specified
Mismetti *et al* (2003)^21^ (open randomised study)	59	Port	Subclavian+ jugular	Nadroparin 2850U or warfarin 1 mg	90 days	Venogram	Nadroparin 6/21 (29%) warfarin 4/24 (17%)	Nadroparin 1/21 (5%) Warfarin 2/24 (8%)
Couban *et al* (2005)^22^ (placebo RCT)	255	Port+ External	Sublavian+ jugular	Warfarin 1 mg or placebo	Catheter duration	Ultrasound± venogram	Symptomatic only	Warfarin 6/130 (5%) Placebo 5/125 (4%)
Verso *et al* (2005)^23^ (placebo RCT)	385	External	Sublavian+ jugular	Enoxaparin 40 mg or placebo	42 days	Venogram	Enoxaparin 22/155 (14%) Placebo 28/155 (18%)	Enoxaparin 2/155 (1%) Placebo 6/155 (3%)

## References

[bib1] Altinbas M, Coskun HS, Er O, Ozkan M, Eser B, Unal A, Cetin M, Soyuer S (2004) A randomized clinical trial of combination chemotherapy with and without low-molecular-weight heparin in small cell lung cancer. J Thromb Haemost 2: 1266–1271 15304029 10.1111/j.1538-7836.2004.00871.x

[bib2] Ambrus JL, Ambrus CM, Mink IB, Pickren JW (1975) Causes of death in cancer patients. J Med 6: 61–64 1056415

[bib3] Baglin TP, Boughton BJ (1986) Central venous thrombosis due to bolus injections of antileukaemic chemotherapy. Br J Haematol 63: 606–607 3730291 10.1111/j.1365-2141.1986.tb07541.x

[bib4] Bauer KA, Rosenberg RD (1987) The pathophysiology of the prethrombotic state in humans: insights gained from studies using markers of hemostatic system activation. Blood 70: 343–350 3607275

[bib5] Bern MM, Lokich JJ, Wallach SR, Bothe Jr A, Benotti PN, Arkin CF, Greco FA, Huberman M, Moore C (1990) Very low doses of warfarin can prevent thrombosis in central venous catheters. A randomized prospective trial. Ann Intern Med 112: 423–428 2178534 10.7326/0003-4819-76-3-112-6-423

[bib6] Bick RL (1978) Alterations of hemostasis associated with malignancy: etiology, pathophysiology, diagnosis and management. Semin Thromb Hemost 5: 1–26 705351 10.1055/s-0028-1087142

[bib7] Bona RD, Hickey AD, Wallace DM (1997) Efficacy and safety of oral anticoagulation in patients with cancer. Thromb Haemost 78: 137–140 9198143

[bib8] Boraks P, Seale J, Price J, Bass G, Ethell M, Keeling D, Mahendra P, Baglin T, Marcus R (1998) Prevention of central venous catheter associated thrombosis using minidose warfarin in patients with haematological malignancies. Br J Haematol 101: 483–486 9633891 10.1046/j.1365-2141.1998.00732.x

[bib9] Bozzetti F, Scarpa D, Terno G, Scotti A, Ammatuna M, Bonalumi MG, Ceglia E (1983) Subclavian venous thrombosis due to indwelling catheters: a prospective study on 52 patients. JPEN J Parenter Enteral Nutr 7: 560–562 6418913 10.1177/0148607183007006560

[bib10] Broviac JW, Cole JJ, Scribner BH (1973) A silicone rubber atrial catheter for prolonged parenteral alimentation. Surg Gynecol Obstet 136: 602–606 4632149

[bib11] Carr KM, Rabinowitz I (2000) Physician compliance with warfarin prophylaxis for central venous catheters in patients with solid tumors. J Clin Oncol 18: 3665–3667 11054439 10.1200/JCO.2000.18.21.3665

[bib12] Cortelezzi A, Moia M, Falanga A, Pogliani EM, Agnelli G, Bonizzoni E, Gussoni G, Barbui T, Mannucci PM (2005) Incidence of thrombotic complications in patients with haematological malignancies with central venous catheters: a prospective multicentre study. Br J Haematol 129: 811–817 15953009 10.1111/j.1365-2141.2005.05529.x

[bib13] Couban S, Goodyear M, Burnell M, Dolan S, Wasi P, Barnes D, Macleod D, Burton E, Andreou P, Anderson DR (2005) Randomized placebo-controlled study of low-dose warfarin for the prevention of central venous catheter-associated thrombosis in patients with cancer. J Clin Oncol 23: 4063–4069 15767639 10.1200/JCO.2005.10.192

[bib14] Craft PS, May J, Dorigo A, Hoy C, Plant A (1996) Hickman catheters: left-sided insertion, male gender, and obesity are associated with an increased risk of complications. Aust NZ J Med 26: 33–39 10.1111/j.1445-5994.1996.tb02904.x 8775526

[bib15] De Cicco CM, Matovic M, Balestreri L, De AV, Fracasso A, Morassut S, Coran F, Babare R, Buonadonna A, Testa V (1995) Antithrombin III deficiency as a risk factor for catheter-related central vein thrombosis in cancer patients. Thromb Res 78: 127–137 7482430 10.1016/0049-3848(95)00041-0

[bib16] Eastridge BJ, Lefor AT (1995) Complications of indwelling venous access devices in cancer patients. J Clin Oncol 13: 233–238 7799025 10.1200/JCO.1995.13.1.233

[bib17] Geerts WH, Pineo GF, Heit JA, Bergqvist D, Lassen MR, Colwell CW, Ray JG (2004) Prevention of venous thromboembolism: the Seventh ACCP Conference on Antithrombotic and Thrombolytic Therapy. Chest 126: 338S–400S 15383478 10.1378/chest.126.3_suppl.338S

[bib18] Gould JR, Carloss HW, Skinner WL (1993) Groshong catheter-associated subclavian venous thrombosis. Am J Med 95: 419–423 8213875 10.1016/0002-9343(93)90312-d

[bib19] Heaton DC, Han DY, Inder A (2002) Minidose (1 mg) warfarin as prophylaxis for central vein catheter thrombosis. Intern Med J 32: 84–88 11885848

[bib20] Heit JA, Silverstein MD, Mohr DN, Petterson TM, O'Fallon WM, Melton III LJ (2000) Risk factors for deep vein thrombosis and pulmonary embolism: a population-based case–control study. Arch Intern Med 160: 809–815 10737280 10.1001/archinte.160.6.809

[bib21] Hickman RO, Buckner CD, Clift RA, Sanders JE, Stewart P, Thomas ED (1979) A modified right atrial catheter for access to the venous system in marrow transplant recipients. Surg Gynecol Obstet 148: 871–875 109934

[bib22] Horattas MC, Wright DJ, Fenton AH, Evans DM, Oddi MA, Kamienski RW, Shields EF (1988) Changing concepts of deep venous thrombosis of the upper extremity – report of a series and review of the literature. Surgery 104: 561–567 3046028

[bib23] Horne III MK, May DJ, Alexander HR, Steinhaus EP, Whitman ED, Chang RC, Doppman JL (1995) Venographic surveillance of tunneled venous access devices in adult oncology patients. Ann Surg Oncol 2: 174–178 7728573 10.1007/BF02303635

[bib24] Kakkar AK, Levine MN, Kadziola Z, Lemoine NR, Low V, Patel HK, Rustin G, Thomas M, Quigley M, Williamson RC (2004) Low molecular weight heparin, therapy with dalteparin, and survival in advanced cancer: the fragmin advanced malignancy outcome study (FAMOUS). J Clin Oncol 22: 1944–1948 15143088 10.1200/JCO.2004.10.002

[bib25] Kearon C (2003) Natural history of venous thromboembolism. Circulation 107: I22–I30 12814982 10.1161/01.CIR.0000078464.82671.78

[bib26] Kerr TM, Lutter KS, Moeller DM, Hasselfeld KA, Roedersheimer LR, McKenna PJ, Winkler JL, Spirtoff K, Sampson MG, Cranley JJ (1990) Upper extremity venous thrombosis diagnosed by duplex scanning. Am J Surg 160: 202–206 2382774 10.1016/s0002-9610(05)80307-1

[bib27] Klerk CP, Smorenburg SM, Buller HR (2003) Thrombosis prophylaxis in patient populations with a central venous catheter: a systematic review. Arch Intern Med 163: 1913–1921 12963564 10.1001/archinte.163.16.1913

[bib28] Klerk CP, Smorenburg SM, Otten HM, Lensing AW, Prins MH, Piovella F, Prandoni P, Bos MM, Richel DJ, van TG, Buller HR (2005) The effect of low molecular weight heparin on survival in patients with advanced malignancy. J Clin Oncol 23: 2130–2135 15699479 10.1200/JCO.2005.03.134

[bib29] Krauth D, Holden A, Knapic N, Liepman M, Ansell J (1987) Safety and efficacy of long-term oral anticoagulation in cancer patients. Cancer 59: 983–985 3815277 10.1002/1097-0142(19870301)59:5<983::aid-cncr2820590522>3.0.co;2-o

[bib30] Lee AY (2005) Management of thrombosis in cancer: primary prevention and secondary prophylaxis. Br J Haematol 128: 291–302 15667530 10.1111/j.1365-2141.2004.05292.x

[bib31] Lee AY, Rickles FR, Julian JA, Gent M, Baker RI, Bowden C, Kakkar AK, Prins M, Levine MN (2005) Randomized comparison of low molecular weight heparin and coumarin derivatives on the survival of patients with cancer and venous thromboembolism. J Clin Oncol 23: 2123–2129 15699480 10.1200/JCO.2005.03.133

[bib32] Levine M, Hirsh J, Gent M, Arnold A, Warr D, Falanga A, Samosh M, Bramwell V, Pritchard KI, Stewart D (1994) Double-blind randomised trial of a very-low-dose warfarin for prevention of thromboembolism in stage IV breast cancer. Lancet 343: 886–889 7908358 10.1016/s0140-6736(94)90008-6

[bib33] Magagnoli M, Masci G, Castagna L, Bramanti S, Morenghi E, Carnaghi C, Santoro A (2005) High incidence of haemostatic interference in cancer patients treated with FOLFOX regimen and concomitant minidose of warfarin. Br J Haematol 129: 709–710 15916696 10.1111/j.1365-2141.2005.05523.x

[bib34] Marie I, Levesque H, Cailleux N, Primard E, Peillon C, Watelet J, Courtois H (1998) Deep venous thrombosis of the upper limbs. Apropos of 49 cases. Rev Med Intern 19: 399–408 10.1016/s0248-8663(98)80864-3 9775181

[bib35] Marinella MA, Kathula SK, Markert RJ (2000) Spectrum of upper-extremity deep venous thrombosis in a community teaching hospital. Heart Lung 29: 113–117 10739487

[bib36] Masci G, Magagnoli M, Zucali PA, Castagna L, Carnaghi C, Sarina B, Pedicini V, Fallini M, Santoro A (2003) Minidose warfarin prophylaxis for catheter-associated thrombosis in cancer patients: can it be safely associated with fluorouracil-based chemotherapy? J Clin Oncol 21: 736–739 12586814 10.1200/JCO.2003.02.042

[bib37] Mismetti P, Mille D, Laporte S, Charlet V, Buchmuller-Cordier A, Jacquin JP, Fournel P, Dutrey-Dupagne C, Decousus H (2003) Low-molecular-weight heparin (nadroparin) and very low doses of warfarin in the prevention of upper extremity thrombosis in cancer patients with indwelling long-term central venous catheters: a pilot randomized trial. Haematologica 88: 67–73 12551829

[bib38] Monreal M, Alastrue A, Rull M, Mira X, Muxart J, Rosell R, Abad A (1996) Upper extremity deep venous thrombosis in cancer patients with venous access devices – prophylaxis with a low molecular weight heparin (Fragmin). Thromb Haemost 75: 251–253 8815570

[bib39] Monreal M, Raventos A, Lerma R, Ruiz J, Lafoz E, Alastrue A, Llamazares JF (1994) Pulmonary embolism in patients with upper extremity DVT associated to venous central lines – a prospective study. Thromb Haemost 72: 548–550 7878630

[bib40] Nightingale CE, Norman A, Cunningham D, Young J, Webb A, Filshie J (1997) A prospective analysis of 949 long-term central venous access catheters for ambulatory chemotherapy in patients with gastrointestinal malignancy. Eur J Cancer 33: 398–403 9155523 10.1016/s0959-8049(97)89012-0

[bib41] Oger E (2000) Incidence of venous thromboembolism: a community-based study in Western France. EPI-GETBP Study Group. Groupe d'Etude de la Thrombose de Bretagne Occidentale. Thromb Haemost 83: 657–660 10823257

[bib42] Piccioli A, Prandoni P, Ewenstein BM, Goldhaber SZ (1996) Cancer and venous thromboembolism. Am Heart J 132: 850–855 8831376 10.1016/s0002-8703(96)90321-x

[bib43] Poller L, McKernan A, Thomson JM, Elstein M, Hirsch PJ, Jones JB (1987) Fixed minidose warfarin: a new approach to prophylaxis against venous thrombosis after major surgery. Br Med J (Clin Res Ed) 295: 1309–1312 10.1136/bmj.295.6609.1309 PMC1248380 3120989

[bib44] Tesselaar ME, Ouwerkerk J, Nooy MA, Rosendaal FR, Osanto S (2004) Risk factors for catheter-related thrombosis in cancer patients. Eur J Cancer 40: 2253–2259 15454250 10.1016/j.ejca.2004.06.023

[bib45] Verso M, Agnelli G, Bertoglio S, Di Somma FC, Paoletti F, Ageno W, Bazzan M, Parise P, Quintavalla R, Naglieri E, Santoro A, Imberti D, Soraru M, Mosca S (2005) Enoxaparin for the prevention of venous thromboembolism associated with central vein catheter: a double-blind, placebo-controlled, randomized study in cancer patients. J Clin Oncol 23: 4057–4062 15767643 10.1200/JCO.2005.06.084

